# Antiglycoxidative properties of amantadine – a systematic review and comprehensive in vitro study

**DOI:** 10.1080/14756366.2022.2137161

**Published:** 2022-11-02

**Authors:** Miłosz Nesterowicz, Małgorzata Żendzian-Piotrowska, Jerzy Robert Ładny, Anna Zalewska, Mateusz Maciejczyk

**Affiliations:** a Students’ Scientific Club “Biochemistry of Civilization Diseases” at the Department of Hygiene, Epidemiology and Ergonomics, Medical University of Bialystok, Białystok, Poland; b Department of Hygiene, Epidemiology and Ergonomics, Medical University of Bialystok, Białystok, Poland; c 1st Department of General Surgery and Endocrinology, Medical University of Bialystok, Białystok, Poland; d Independent Laboratory of Experimental Dentistry, Medical University of Bialystok, Białystok, Poland

**Keywords:** Amantadine, protein glycation, oxidative stress, carbonyl stress

## Abstract

An important drug used in the treatment of Parkinson’s disease is amantadine. We are the first to perform a comprehensive study based on various glycation and oxidation factors, determining the impact of amantadine on protein glycoxidation. Sugars (glucose, fructose, galactose) and aldehydes (glyoxal, methylglyoxal) were used as glycation agents, and chloramine T was used as an oxidant. Glycoxidation biomarkers in albumin treated with amantadine were generally not different from the control group (glycation/oxidation factors), indicating that the drug did not affect oxidation and glycation processes. Molecular docking analysis did not reveal strong binding sites of amantadine on the bovine serum albumin structure. Although amantadine poorly scavenged hydroxyl radical and hydrogen peroxide, it had significantly lower antioxidant and antiglycation effect than all protein oxidation and glycation inhibitors. In some cases, amantadine even demonstrated glycoxidant, proglycation, and prooxidant properties. In summary, amantadine exhibited weak antioxidant properties and a lack of antiglycation activity.

## Graphical Abstract

**Figure:**
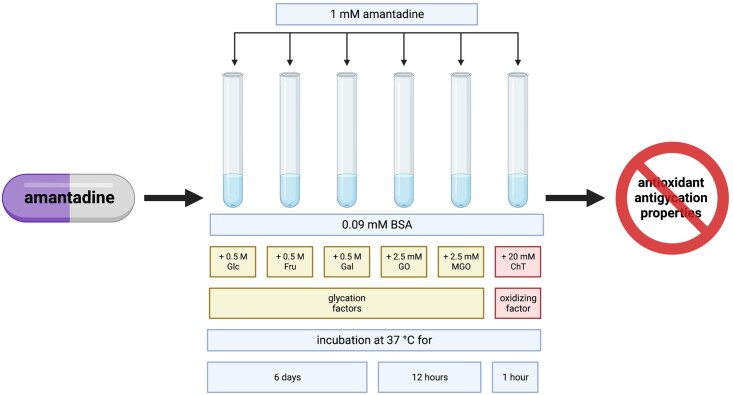

## Introduction

The pathogenesis of several systemic disorders is related to the overproduction of reactive oxygen (ROS) and nitrogen (RNS) species. Increased ROS/RNS formation occurs under the influence of external factors (e.g. diet, xenobiotics, ionising radiation) as well as cellular metabolic disorders (e.g. hyperglycaemia, hypercholesterolaemia, and obesity)^1^^,^^2^. When the redox balance shifts in favour of oxidative reactions, cell metabolism is disrupted, which is defined as oxidative stress. This process plays a crucial role in neurodegenerative disorders such as Parkinson’s disease, Alzheimer’s disease, and amyotrophic lateral sclerosis^3–5^. Consuming significant amounts of oxygen, the brain is particularly vulnerable to oxidative stress. Neuronal cell membranes are rich in polyunsaturated fatty acids, making them highly susceptible to oxidation by ROS. The brain is also marked by deposition of redox-active metals and low activity of antioxidant enzymes, both increasing with age^6^. Thus, it is not surprising that neuronal proteins, lipids, and DNA undergo oxidative injury during ROS-mediated cerebral neurodegeneration. Autoxidation of neurotransmitters, like dopamine or hydroquinones, is not the only source of free radicals in the brain. The main generator of ROS in neurons/glial cells is NADPH oxidase (NOX) activated by a receptor for advanced glycation end products (AGE)^7^. AGE stimulate the nuclear factor kappa-light-chain-enhancer of activated B cells (NF-κB) pathway, enhancing the expression of interleukin 1β (IL-1β), interleukin 6 (IL-6), and tumour necrosis factor α (TNF-α), as well as cerebral adhesion molecules, e.g. intercellular adhesion molecule 1 (ICAM-1), vascular cell adhesion molecule 1 (VCAM-1), and monocyte chemoattractant protein 1 (MCP-1)^7^. AGE also promote ceramide synthesis and β-amyloid (βA) accumulation, leading to neuronal death via apoptosis and necrosis^8^^,^^9^. Interestingly, βA can mediate the phosphorylation of mitogen-activated protein kinase (MAPK) via AGE signalling. Activation of MAPK pathway causes synapse decline, thus triggering adverse changes in memory and study processes^10^. Therefore, compounds with antioxidant and antiglycation activity can be sought to treat neurodegenerative diseases. Strategies to prevent protein glycation include endogenous defence mechanisms as well as synthetic and natural inhibitors. The basis of the body’s protection is the action of enzymes (such as the glyoxalase I and II) which prevent or inhibit glycation as well as participate in the repair of damaged proteins. Synthetic substances can act mainly by affecting ROS formation, binding sugars to proteins, reducing the formation of Amadori products (AP)/late products of protein glycation, and breaking AGE-protein cross-links^11^^,^^12^. Phytonutrients such as anthocyanins have a similar effect. Additionally, they are fairly safe, inexpensive and can be administered orally. Antioxidants (e.g. catalase [CAT] and superoxide dismutase [SOD]) also present antiglycation properties; however, they can undergo the glycation process, which can be prevented by thymoquinone^13^^,^^14^.

**Figure 1.**
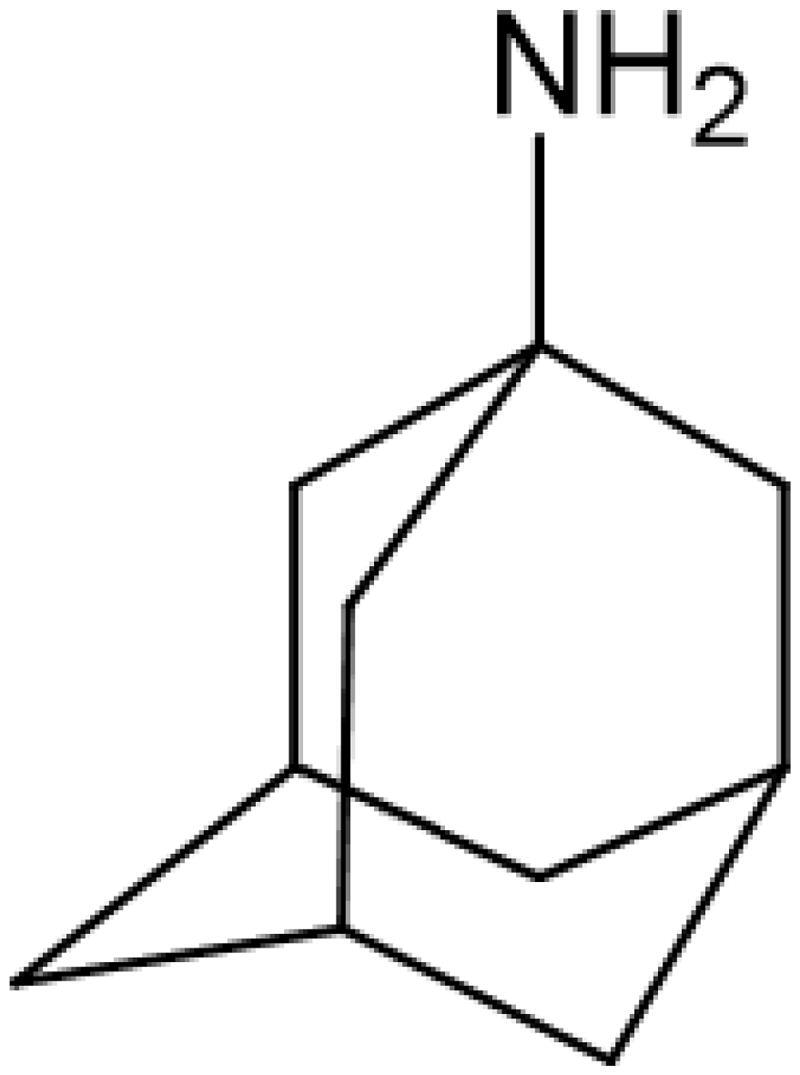
Chemical formula of amantadine.

Amantadine (C_10_H_17_N; 1-adamantanamine; Figure 1) is a synthetic tricyclic amine, a derivative of adamantane^15^. It is an antidyskinetic drug used in Parkinson’s disease and Parkinsonian syndromes, as well as in the prevention/treatment of influenza A^16^. Although the mechanism of the drug’s action is not precisely known, amantadine increases the extracellular concentration of dopamine by enhancing its release in the striatum and blocking its reuptake by presynaptic neurons^17^. Dopamine regulates motor activity and cognitive function like memory and learning^18^. Loss of dopaminergic cells is the essence of the Parkinson’s disease^19^. Pharmacotherapy of Parkinson’s disease requires the use of levodopa, which is a precursor of dopamine, as well as dopamine receptor agonists (e.g. ropinirole and pramipexole)^20^.

Amantadine is also a low-affinity antagonist of N-methyl-D-aspartate (NMDA) glutamate receptor subtype. Amantadine inhibits the release of acetylcholine through NMDA receptors, exhibiting anticholinergic effect^21^. It should be noted that NMDA antagonists, even in low concentrations, trigger the underactivation of brain NMDA receptors. This results in learning and memory disorders, psychosis and, ultimately, excitotoxic damage to neurons. The NMDA receptor system also gradually weakens with age. Oxidative stress and βA accumulation can exacerbate the hypofunction of the receptor, leading to extensive neurodegeneration^22^.

On the other hand, amantadine has a virostatic effect, inhibiting the early stages of viral replication by blocking the proton pump of viral M2 protein, stopping the removal of the viral envelope, and inactivating newly synthesised viral haemagglutinin^23^^,^^24^. Effects on late stages of replication have been found for representative avian influenza viruses^24^.

Not all mechanisms of the pharmacological action of amantadine are well understood. Since amantadine has been successfully used in oxidative stress-related Parkinson’s disease, its therapeutic activity may be based on preventing protein glycoxidation^25^^,^^26^. Recently, the potential use of amantadine has been postulated in patients with SARS-CoV infection which occurs with redox imbalance and inflammation^27–34^. However, data on the effect of amantadine on carbonyl stress are inconclusive. It was shown that amantadine reduced lipid peroxidation as well as scavenged the superoxide anion (O_2_•^−^) and hydroxyl radical (HO•) generation in *in vitro* models^35–38^. In vivo, the drug counteracted oxidative damage in 2-deoxyribose and dopamine-generating neurons, increased reduced glutathione (GSH) production, and alleviated lipid peroxidation^38–42^. However, there are no studies evaluating the antiglycation properties of amantadine. Therefore, we are the first to investigate amantadine for its both antioxidant and antiglycation activity.

## Materials and methods

### Systematic review

The review of the literature was conducted between 1995 and May 2022 on Medline (PubMed) database. The accessible bibliography was searched using keywords: [amantadine and antiglycoxidative properties], [amantadine and antiglycation properties] [amantadine and antioxidative properties], [amantadine and oxidative stress], [amantadine and carbonyl stress], [amantadine and protein glycation], [amantadine and nitrosative stress], and [amantadine and ROS scavenging]. Inclusion and exclusion criteria are presented in Table 1.

**Table 1.** Inclusion and exclusion criteria.

Inclusion criteria	Exclusion criteria
Publications only in English	Publications in other languages
Articles describing the antiglycoxidative activity of amantadine	Articles not describing the antiglycoxidative activity of amantadine
Results collected during human research and also experimental in vivo and in vitro studies	Surveys, review articles, as well as case reports

The initial data was explored by evaluating titles and abstracts of publications independently by two investigators (M.N., M.M.). Next, the other two authors scrutinised all previously extracted manuscripts (M.Z.P., A.Z.). The papers compliant with the inclusion and exclusion criteria were utilised for the final analysis. The researchers’ level of reliability was evaluated with Cohen’s kappa coefficient (κ) which equalled κ = 0.94. All articles were assessed methodologically, and the following factors were analysed: authors, year of publication, study design, size of the experiment population, inclusion and exclusion criteria, research length, and endpoints.

### Reagents and equipment

All chemicals (analytical grade) were purchased from Sigma-Aldrich (Nümbrecht, Germany/Saint Louis, MO, USA). Directly before use, solutions were sterilised by filtration through 0.2-mm-membrane filters. The absorbance and fluorescence were assessed with an Infinite M200 PRO multimode microplate reader (Tecan Group Ltd., Männedorf, Switzerland).

### Scavenging of reactive oxygen species (ROS)

#### Hydroxyl radical (HO•) scavenging

The scavenging activity of HO• was measured via the modified assay described by Su et al.^43^ In brief, 0.25 mL of ferrous sulphate (8 mM), 0.4 mL of hydrogen peroxide (6 mM), 0.25 mL of distilled water, 0.5 mL of the samples (final concentration: 1 mM), and 0.2 mL of sodium salicylate (20 mM) were mixed and incubated at 37 °C for 1 h. The absorbance of the reaction mixture was measured at 562 nm wavelength. The scavenged HO• (%) was counted by the formula: [1 – {(*A*_1_ – *A*_2_)/*A*_0_}] × 100%, where *A*_0_ represents the absorbance of the control (without additives), *A*_1_ – after the addition of the drugs, and *A*_2_ – without sodium salicylate^43^.

#### Hydrogen peroxide (H_2_O_2_) scavenging

The assessment of H_2_O_2_ scavenging activity was performed in compliance with the method by Kwon et al.^44^ Firstly, butylated hydroxytoluene (BHT) (87.3 mg), sulphuric acid (H_2_SO_4_) (10 μL), xylenol orange (7.6 mg) and ferrous ammonium sulphate (10 mg) were mixed in 100 mL of 90% methanol-water solution in order to obtain ferrous ion oxidation-xylenol orange (FOX). Then, 50 mM of H_2_O_2_ and the samples (final concentration: 1 mM) were mixed (1:1, v/v) and incubated at room temperature for 30 min. Next, 10 μL of high-performance liquid chromatography (HPLC)-grade methanol was added to 90 μL of the sample solution in H_2_O_2_. After that, 0.9 mL of the FOX reagent was added to the above mixture and it was then vortexed and incubated at room temperature for 30 min. The absorbance of the reaction product, ferric-xylenol orange complex, was assayed spectrophotometrically at 560 nm wavelength. The scavenged H_2_O_2_ (%) was calculated according to the formula: [1 – {(*A*_1_ – *A*_2_)/*A*_0_}] × 100%, where *A*_0_ is the absorbance of the control (without additives), *A*_1_ – after the addition of the drugs, and *A*_2_ – without the FOX reagent^44^.

### Redox status

#### 2,2-Diphenyl-1-picrylhydrazyl (DPPH) radical scavenging capacity

The determination of free radical scavenging activity was performed based on the decolourisation of the DPPH radical according to Kwon et al.^44^ Briefly, 30 µL of the diluted sample was added to 180 µL of DPPH solution (0.13 mg/mL). Next, the mixture was replenished with methanol to a final volume of 210 µL. The DPPH solution was used as a control. After that, the absorbance of the reaction mixture, incubated for 20 min, was measured at 517 nm. The inhibition rate was presented as the percentage of DPPH radical elimination and counted according to the formula: [(*A*_blank_ – *A*_sample_)/*A*_blank_] × 100%, where *A*_blank_ is the absorbance of the blank DPPH solution, and *A*_sample_ – DPPH solution after the addition of the sample^44^.

#### Total antioxidant capacity (TAC)

TAC assessment was performed with the Erel’s method^45^ based on the ability to neutralise 2,2-azino-bis-(3-ethylbenzothiazoline-6-sulphonate (ABTS) cationic radical under the influence of antioxidants contained in the sample. ABTS•+ was received through a reaction of ABTS with potassium persulphate and incubation at room temperature for 12 h. 10 μL of samples were mixed with 1 mL of ABTS•+. The absorbance was measured at 660 nm. The concentration of TAC was read from the standard curve for 6-hydroxy-2,5,7,8-tetramethylchroman-2-carboxylic acid (Trolox)^45^.

#### Total oxidant status (TOS)

The Erel’s method was used to calculate TOS^46^. In this test, disparate oxidants cause the transformation of the ferrous ion-*o*-dianisidine complex into the ferric ion. The ferric ion in an acidic medium forms a coloured complex with xylenol orange. 35 μL of the sample, 225 μL of reagent 1 (150 μM xylenol orange, 140 mM NaCl and also 1.35 glycerol in 25 mM H_2_SO_4_ solution, pH 1.75) as well as 11 μL of reagent 2 (5 mM ferrous ion with 10 mM *o*-dianisidine in 25 mM H_2_SO_4_ solution), were mixed. The wavelength of 560/800 nm was used to measure the absorbance. The TOS level was read from H_2_O_2_ standard curve^46^.

### Bovine serum albumin (BSA) model

The glycation and/or oxidation of BSA was conducted in compliance with a previously published method^47–52^. Immediately, BSA of 96% purity was dissolved in sodium phosphate buffer (0.1 M, pH 7.4) containing 0.02% sodium azide (as a preservative). As glycation agents, both sugars – glucose (Glc), fructose (Fru) and galactose (Gal) as well as aldehydes – glyoxal (GO) and methylglyoxal (MGO) – were used. To measure the effects of additives on the process of protein glycation, BSA was incubated with 1 mM amantadine and 0.5 M Glc, Fru, and Gal for six days, or 2.5 mM GO and MGO for 12 h^48^^,^^51^^,^^53–55^. GO, and MGO were used within a month after delivery, and working solutions were prepared briefly before assessment^47^. For measurements of the impact of additives on protein oxidation, BSA with amantadine was incubated with 20 mM chloramine T (ChT) for an hour^56^. Every sample was incubated in the dark, in closed vials with continuous shaking (50 rpm) at 37 °C^47^^,^^48^^,^^51^. The incubation mixtures included BSA at a final concentration of 0.09 mM.

Glycation agent concentrations and the optimal incubation conditions for studies on the modification of the glycoxidation rate by additives were assayed and validated according to the previous kinetic studies^47^^,^^48^. Despite the fact that the concentrations of oxidants, sugars and aldehydes were much higher than their physiological levels, they are useful for modelling in a comparatively short time, the physiological processes occurring in the human body over weeks or even months^47^^,^^48^^,^^51^. Such experimental conditions are applied routinely to determine antiglycation properties of new substances^47–49^^,^^51^^,^^53^^,^^56–58^.

To compare the results obtained for amantadine, aminoguanidine was used as a known protein oxidation inhibitor, and α-lipoic acid (ALA), N-acetylcysteine (NAC), and ascorbic acid (AA) – as antioxidants^47–52^. The level of all additives was 1 mM and it was determined in accordance with the other *in vitro* studies, proportionally to the high concentrations of glycation agents^47–49^^,^^51^^,^^53^^,^^56–58^. The study was conducted in three series, every time in duplicate.

### Protein glycoxidation products

Tryptophan (TRY), kynurenine (KN), N-formylkynurenine (NFK), and dityrosine (DT) were assessed using fluorescence emission and excitation at 95/340, 365/480, 325/434, and 330/415 nm wavelengths, respectively. Before the measurement, the investigated solutions were diluted with 0.1 M H_2_SO_4_ (1:5, v/v). Results were standardised based on fluorescence of 0.1 mg/mL quinine sulphate in 0.1 M H_2_SO_4_^59^^,^^60^_._

### Protein glycation products

#### Amadori products (AP)

The total amount of AP was assessed by means of a colourimetric Nitro Blue Tetrazolium (NBT) assay. The absorbance was estimated at 525 nm wavelength with the use of monoformazan extinction coefficient (12 640 M^−1^cm^−1^)^61^.

#### β-amyloid (βA)

Thioflavin T evaluation was performed to mark fluorescence emitted at the moment of binding of amyloid fibrils or oligomers to thioflavin T. 90 μL of samples were mixed with 10 μL of thioflavin T and placed on a microplate. The intensity of fluorescence was calculated at 385/485 nm wavelength^62^^,^^63^.

#### Advanced glycation end products (AGE)

The content of AGE was assayed spectrofluorometrically. AGE-specific fluorescence was marked at 440/370 nm wavelength in a 96-well microplate reader^64^^,^^65^. Before the reading, samples were diluted with PBS (1:5, v/v). The AGE level was also measured using the commercial ELISA method (USCN, Life Science, Wuhan, China), according to the manufacturer’s instructions.

### Protein oxidation products

#### Protein carbonyls (PC)

Assessment of PC concentration was conducted with the use of the reaction of carbonyls with 2,4-dinitrophenylhydrazine (DNPH) in proteins damaged by oxidation. Reaction product absorbance was marked colourimetrically at 355 nm wavelength. The absorption coefficient for 2,4-DNPH (22 000 M^−1^cm^−1^) was used as a standard^66^.

#### Advanced oxidation protein products (AOPP)

To examine the level of AOPP, a spectrophotometric assay was conducted. 200 μL of the investigated samples diluted with PBS in a 1:5 ratio (v/v), the standard solutions (0–100 μmol/L), and 200 μL of blank PBS solution were placed on a 96-well microplate. Then, 10 μL of 1.16 M potassium iodide and 20 μL of acetic acid were added to the wells. The absorbance was determined instantly in a microplate reader at 340 nm wavelength in comparison with the blank solution (200 μL PBS, 10 μL potassium iodide, 20 μL acetic acid). ChT solutions showed linear absorbance in the range of 0–100 μmol/L^64^.

### Molecular docking analysis

Molecular docking is used in the in silico method of predicting the preferred position of a ligand after binding to a macromolecule (e.g. protein). BSA was used as a receptor in an interaction study with the amantadine molecule. Possible attachment of the drug to BSA could suggest a mechanism of protective action against carbonyl stress. A three-dimensional structure of BSA (PDB ID: 4F5S)^67^ was downloaded from the website of Protein Data Bank (PDB) (https://www.rcsb.org/) in the .pdb format. It was the crystal structure established with the X-ray diffraction method at a 2.47 Å resolution value^67^. The three-dimensional structure of amantadine (PubChem CID: 2130)^68^ was obtained from the National Library of Medicine website (https://pubchem.ncbi.nlm.nih.gov/) as a .sdf file. The BSA molecule was processed using AutoDock MGL Tools^69^ through the deletion of all water particles and addition of polar hydrogens and Kollman charges in order to minimise the energy. The prepared protein structure was saved in a .pdbqt format. Molecular docking simulation was performed by AutoDock Vina^70^ with the grid size of 40 × 40 × 40. The grid box had a 0.375 Å spacing located in x, y, and z centres: 34.885, 23.976, and 98.792, respectively. The exhaustiveness parameter value was determined at the level of 8. PyMOL 2.5 was used to visualise molecular docking^71–74^.

### Statistical analysis

The results were shown as a percentage of the respective control values (BSA + glycation (Glc, Fru, Gal, GO, MGO)/oxidising agent [ChT]). Differences between groups were assayed using the one-way analysis of variance (ANOVA) followed by Tukey’s *post hoc* test for multiple comparisons, and *p* < 0.05 was allowed to be statistically significant. Multiplicity adjusted *p* values was also evaluated. The statistical analysis was performed with GraphPad Prism 9 (GraphPad Software, La Jolla, CA, USA).

## Results

### Systematic review

**Figure 2.**
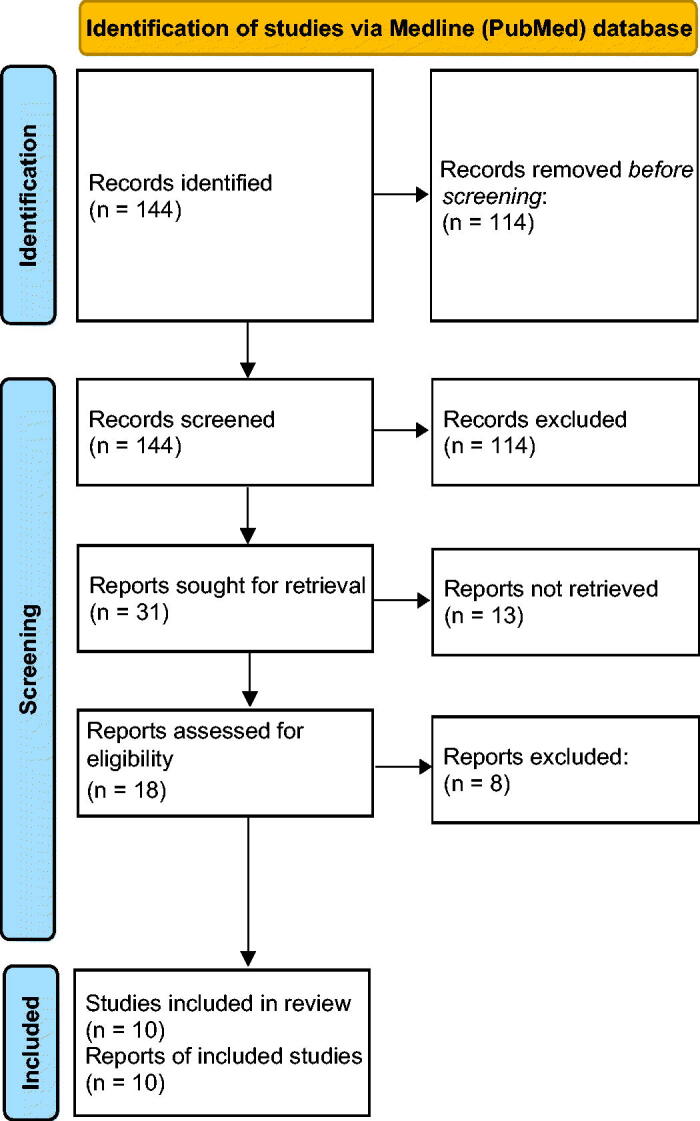
Flow diagram of systematic review methodology (Prisma).

Our systematic review of the bibliography identified 144 publications from the Medline (PubMed) database, from which 113 were excepted because of the title. Out of 31 read abstracts, 18 complied with the inclusion and exclusion criteria. Out of the eligible works, eight were found not to be related to the subject of our research. However, 10 papers were finally included (Figure 2). The results of our systematic review are presented in Table 2.

**Table 2.** Multidirectional properties of amantadine in clinical and experimental studies.

Study design	Endpoints	References
In vitro studies		
Cells derived from rat livers (cytochrome P450 system) and whole blood treated with amantadine solutions (1-1000 μm)	Amantadine decreased lipid peroxidation and whole blood chemiluminescence, but only in 1000 μM	^35^
Hypoxanthine/xanthine oxidase (HX/XO) superoxide generating system	Amantadine scavenged HO• and O_2_•^-^	^38^
2-Deoxyribose and dopamine-liberating neurons treated with amantadine solutions (0.05–1.0 mm)	Amantadine prevented oxidative stress-induced damage to neurons, generating 2-deoxyribose and dopamine	^38^
Primary cultures (different composition of neurons, microglia, and astroglia) of rat mesencephalon pre-treated with amantadine solution (30 µm) and treated with lipopolysaccharides (LPS) and 1-methyl-4-phenyl-1,2,3,6-tetrahydropyridine (MPTP) (dopaminergic neurotoxins)	Amantadine showed a protective impact of LPS and MPTP toxicity for rat midbrain cultures by inhibiting the release of proinflammatory factors in microglia, enhancing the astroglial expression of a glial-derived neurotrophic factor (GNDF), and also alleviating activation of Phox	^36^
In vitro ROS scavenging in the neutrophil respiratory burst, hydrogen donating and stabilising DPPH, ABTS•^+^ scavenging, as well as ferric reducing antioxidant power (FRAP) of amantadine solutions	Amantadine did not show in vitro antioxidant capacity in any assay	^75^
In vitro DPPH radical scavenging capacity of amantadine and rasagiline solutions (200–1000 µg/mL)	At lower concentrations (200–400 µg/mL), there was a definite difference between amantadine and rasagiline, with amantadine showing more intense antioxidant activity than rasagiline; at higher doses (600–1000 µg/mL), antioxidant and radical scavenging activities of both drugs were comparable; the experiment proved the intrinsic activity of rasagiline and amantadine, which may alleviate the oxidative stress pathways	^37^
In vivo studies		
Wistar albino rats treated with amantadine (2 mg/mL, twice/day) for a week, a month, or three months; total thiols and malondialdehyde concentrations in rat corneas measured	Amantadine did not change the median levels of total thiols and malondialdehyde in comparison to the control group	^76^
6-Hydroxydopamine (6-OHDA)-induced parkinsonism Wistar albino rat model; animals treated with spirulina (500 mg/kg, once or twice/day) or a combination of spirulina (500 mg/kg, once/day) with amantadine (20 mg/kg, once/day) for 30 days before and 14 days after 6-OHDA injection; post-lesion produced rotational behaviour measurement at two-week intervals (37th and 44th day); locomotors activity assayed on 44th day; muscle coordination assessed on 48th day; antioxidant tests (thiobarbituric acid reactive substances [TBARS] and [GSH]), as well as dopamine level determined on 49th day	Both body rotations (ipsilateral and contralateral) were significantly decreased after treatment with spirulina (twice/day); a higher percentage of improvement was shown in the reduction of both body rotations in the animals administered spirulina + amantadine; body movements and locomotor activity were improved in spirulina + amantadine- and spirulina-treated-twice/day group; similar results were also seen in antioxidant levels which later reached the normal value; the dopamine contents increased only in spirulina + amantadine group	^39^
Spinal cord injury (SCI) Sprague–Dawley rat model treated with amantadine (45 mg/kg/day) for a week; oxidative stress (malondialdehyde and GSH level, as well as myeloperoxidase (MPO) activity), inflammation, and angiogenesis parameters assessed	Amantadine reduces oxidative stress, inflammation, as well as apoptosis and alleviates spinal SCI by inducing angiogenesis	^40^
White rat model of severe traumatic brain injury (TBI) treated with amantadine (5 mg/kg), ademol (2 mL/kg), or 0.9% NaCl solution (2 mL/kg); brain oxidative stress parameters determined	Amantadine significantly reduced lipid peroxidation and oxidative degradation of proteins, as well as enhanced antioxidant enzymes concentration in a damaged brain, thus, it is relevantly more effective compared to 0.9% NaCl solution, but significantly less efficiently in comparison with ademol	^41^
32 patients suffering from TBI of whom 18 treated with amantadine in an open clinical trial	Amantadine-treated patients showed decreased malondialdehyde concentration, increased β-carotene level, and longer survival after only one week of therapy	^42^

*Note:* DPPH: 2,2-diphenyl-1-picrylhydrazyl; FRAP: ferric reducing antioxidant power; GNDF: glial-derived neurotrophic factor; GSH: reduced glutathione; HO•: hydroxyl radical; HX/XO: hypoxanthine/xanthine oxidase; LPS: lipopolysaccharides; MPO: myeloperoxidase; MPTP: 1-methyl-4-phenyl-1,2,3,6-tetrahydropyridine; O_2_•^-^: superoxide anion; ROS: reactive oxygen species; SCI: spinal cord injury; TBARS: thiobarbituric acid reactive substances; TBI: traumatic brain injury; 6-OHDA: 6-hydroxydopamine.

### Scavenging of reactive oxygen species (ROS)

ROS are chemically active molecules formed in enzymatic or non-enzymatic reactions of oxidative metabolism. Although at low concentrations they participate in numerous physiological processes, elevated ROS levels lead to oxidative modifications of cellular biomolecules. Evaluation of the scavenging capacity of HO• and H_2_O_2_ provides information on the antioxidant properties of the test sample^77^^,^^78^.

#### Hydroxyl radical (HO•) scavenging

**Figure 3.**
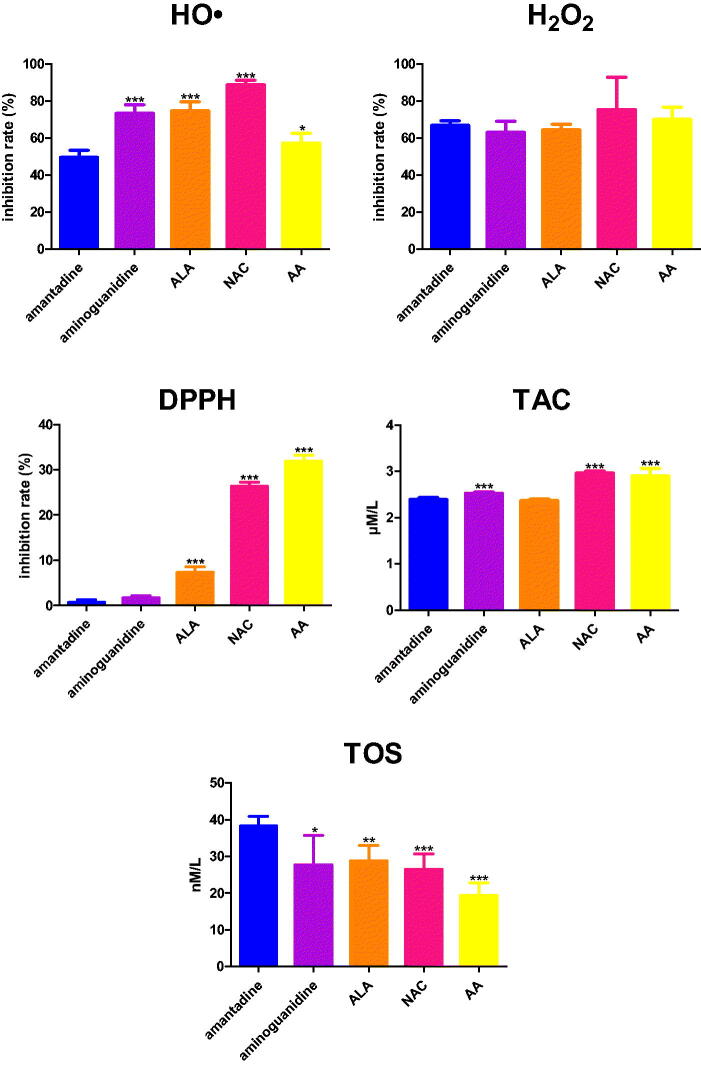
The influence of amantadine and other additives on scavenging of ROS and total antioxidant potential. AA: ascorbic acid; ALA: α-lipoic acid; DPPH: 2,2-diphenyl-1-picrylhydrazyl radical scavenging capacity; H_2_O_2_: hydrogen peroxide scavenging; HO•: hydroxyl radical scavenging; NAC: N-acetylcysteine; TAC: total antioxidant capacity; TOS: total oxidant status; ∗*p* < 0.05 versus control (amantadine); ∗∗*p* < 0.01 versus control (amantadine); ∗∗∗*p* < 0.001 versus control (amantadine).

Amantadine scavenged HO• at 49.6%. The inhibition rate of HO• scavenging of aminoguanidine (+23.9%, *p* < 0.0001), ALA (+25%, *p* < 0.0001), NAC (+39.2%, *p* < 0.0001), as well as AA (+7.7%, *p* = 0.0148) was significantly higher than the inhibition rate of amantadine (Figure 3).

#### Hydrogen peroxide (H_2_O_2_) scavenging

Amantadine scavenged H_2_O_2_ at 66.7%. There were not any significant differences in the inhibition rate of H_2_O_2_ scavenging when compared to the study drug (Figure 3).

### Redox status

The redox status is the balance between oxidants and antioxidants. It has a major impact on various cellular processes. DPPH assessment allows marking the antioxidant potential with the mechanism of a single-electron transfer. TAC is the total capacity to neutralise free radicals. It was shown that synergism of several antioxidants gives a greater antioxidant power than each of the compounds individually. TOS constitutes the total amount of oxidant molecules present in the sample^44–46^^,^^79^.

#### 2,2-Diphenyl-1-picrylhydrazyl (DPPH) radical scavenging capacity

The inhibition rate of DPPH radical scavenging capacity was statistically increased in ALA (+6.6%, *p* < 0.0001), NAC (+25.7%, *p* < 0.0001), and AA (+31.2%, *p* < 0.0001) in comparison with amantadine (Figure 3).

#### Total antioxidant capacity (TAC)

TAC concentration was enhanced compared to amantadine in the case of aminoguanidine (+0.1%, *p* = 0.0001), NAC (+3%, *p* < 0.0001), and AA (+2.9%, *p* < 0.0001) (Figure 3).

#### Total oxidant status (TOS)

TOS level of aminoguanidine (−10.6%, *p* = 0.0207), ALA (-9.5%, *p* = 0.0018), NAC (−11,8%, *p* = 0.0005), as well as AA (−18.9%, *p* < 0.0001) was significantly higher than its level of amantadine (Figure 3).

### Protein glycoxidation products

Tyrosine (TYR) and TRY are amino acids highly susceptible to glycoxidative damage. The product of the breakdown of TRY in the kynurenine pathway is NFK, which is converted to KN. The cross-linking of two glycoxidised TYR results in DT^80^^,^^81^.

**Figure 4.**
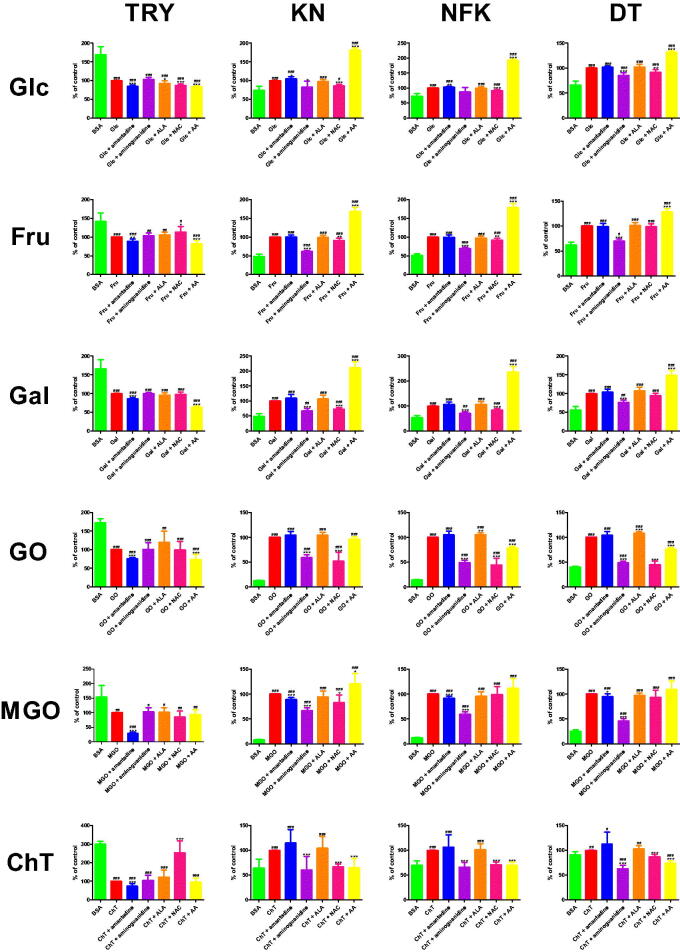
The influence of amantadine and other additives on protein glycoxidation products in various models. AA: ascorbic acid; ALA: α-lipoic acid; BSA: bovine serum albumin; ChT: chloramine T-induced albumin oxidation; DT: dityrosine; Fru: fructose-induced albumin glycation; Gal: galactose-induced albumin glycation; Glc: glucose-induced albumin glycation; GO: glyoxal-induced albumin glycation; KN: kynurenine; MGO: methylglyoxal-induced albumin glycation; NAC: N-acetylcysteine; NFK: N-formylkynurenine; TRY: tryptophan; ∗*p* < 0.05 versus positive control (glycation/oxidising agent); ∗∗*p* < 0.01 versus positive control (glycation/oxidising agent); ∗∗∗*p* < 0.001 versus positive control (glycation/oxidising agent); ^#^*p* < 0.05 versus negative control (BSA); ^##^*p* < 0.01 versus negative control (BSA); ^###^*p* < 0.001 versus negative control (BSA).

The fluorescence of TRY was significantly lower in Glc + amantadine (−15.0%), Glc + ALA (−7.9%), Glc + NAC (−13.4%), and Glc + AA (−16.7%) than in Glc. The parameter was effectively reduced in Glc (−40.8%), Glc + amantadine (−34.7%), Glc + aminoguanidine (−41.8%), Glc + ALA (−37.6%), Glc + NAC (−35.3%), as well as Glc + AA (−34%) in comparison with BSA. TRY content was markedly decreased in Fru + amantadine (−12%) and Fru + AA (−17.8%) versus Fru. This biomarker was substantially enhanced in Fru + NAC (+13.6%) compared to Fru alone. TRY fluorescence was effectively reduced in Fru (−29.4%), Fru + amantadine (−25.8%), Fru + aminoguanidine (−30.4%), Fru + ALA (−31%), Fru + NAC (−33.4%), and Fru + AA (−24.1%) in comparison with BSA. The content of TRY was considerably suppressed in Gal + amantadine (−14.3%) and Gal + AA (−36.7%) versus Gal. This parameter was relevantly lower in Gal (−39.9%), Gal + amantadine (−34.2%), Gal + aminoguanidine (−26.4%), Gal + ALA (−38.1%), Gal + NAC (−39.1%), and Gal + AA (−25.2%) than in BSA. The fluorescence of TRY was significantly attenuated in GO + amantadine (−24.5%) and also GO + AA (−26.9%)compared with GO. The marker was efficiently decreased in GO (−42%), GO + amantadine (−31.7%), GO + aminoguanidine (−42.4%), GO + ALA (−50.3%), GO + NAC (−41.6%), as well as GO + AA (−30.7%) compared to BSA. TRY content was markedly reduced in MGO + amantadine (−70.6%) in comparison with MGO. This biomarker was significantly decreased in MGO (−34.9%), MGO + amantadine (−10.3%), MGO + aminoguanidine (−35.9%), MGO + ALA (−35.6%), MGO + NAC (−29.8%), as well as MGO + AA (−32.6%) when compared to BSA. TRY fluorescence was markedly inhibited in ChT + amantadine (−26.7%) versus ChT. The parameter was relevantly higher in ChT + NAC (+153.9%) than in ChT alone. TRY content was significantly diminished in ChT (−66.7%), ChT + amantadine (−48.9%), ChT + aminoguanidine (−69.2%), ChT + ALA (−81.8%), and ChT + AA (−63.8%) in comparison with BSA (Figure 4).

The content of KN was significantly elevated in Glc + amantadine (+4.8%) and Glc + AA (+81.5%) compared to Glc. The biomarker was effectively reduced in Glc + aminoguanidine (−17.6%) and Glc + NAC (−13.7%) versus Glc. The fluorescence of KN was markedly higher in Glc (+34.6%), Glc + amantadine (+36.3%), Glc + ALA (+33.7%), Glc + NAC (+29.9%), as well as Glc + AA (+62.8%) in comparison with BSA. The parameter was substantially decreased in Fru + aminoguanidine (−38.5%) and Fru + NAC (−8.9%) when compared to Fru. KN fluorescence was effectively increased in Fru + AA (+68.2%) versus Fru alone. The marker was relevantly enhanced in Fru (+106.3%), Fru + amantadine (+106.6%), Fru + aminoguanidine (+65.4%), Fru + ALA (+105%), Fru + NAC (+96.9%), as well as Fru + AA (+178.8%) compared to BSA. KN content was notedly attenuated in Gal + aminoguanidine (−33%) and Gal + NAC (−27.1%) versus Gal. The biomarker was significantly higher in Gal + AA (+111%) than in Gal alone. The fluorescence of KN was effectively intensified in Gal (+103.6%), Gal + amantadine (+113.6%), Gal + aminoguanidine (+69.4%), Gal + ALA (+111%), Gal + NAC (+75.6%), and Gal + AA (+218.6%) compared with BSA. The parameter was markedly lower in GO + aminoguanidine (−40.7%) and GO + NAC (−47.8%) than in GO. KN fluorescence was substantially increased in GO (+677.6%), GO + amantadine (+710.1%), GO + aminoguanidine (+402.1%), GO + ALA (+708.8%), GO + NAC (+353.6%), as well as GO + AA (+655.1%) in comparison with BSA. The marker was significantly reduced in MGO + amantadine (−11.2%), MGO + aminoguanidine (−33.3%), and MGO + NAC (−17%) versus MGO. KN content was notedly increased in MGO + AA (+20%) compared to MGO alone. The biomarker was substantially elevated in MGO (+1148.1%), MGO + amantadine (+1019.5%), MGO + aminoguanidine (+766.1%), MGO + ALA (+1085.1%), MGO + NAC (+953.4%), as well as MGO + AA (+1377.8%) versus BSA. The fluorescence of KN was considerably lower in ChT + aminoguanidine (−39.4%), ChT + NAC (−33.7%), and ChT + AA (−35.4%) than in ChT. The content of KN was relevantly elevated in ChT (+56.3%), ChT + amantadine (+64.7%), and ChT + ALA (+58.8%) in comparison with BSA (Figure 4).

The fluorescence of NFK was significantly elevated in Glc + amantadine (+3.4%) and Glc + AA (+92.6%) compared to Glc. The biomarker was effectively reduced in Glc + NAC (−8.7%) versus Glc. The content of NFK was markedly higher in Glc (+37.1%), Glc + amantadine (+38.3%), Glc + ALA (+36.8%), Glc + NAC (+33.8%), as well as Glc + AA (+71.4%) in comparison to BSA. The parameter was substantially decreased in Fru + aminoguanidine (−30.2%) and Fru + NAC (−8.1%) when compared to Fru. NFK content was effectively increased in Fru + AA (+79%) versus Fru alone. The marker was relevantly enhanced in Fru (+96.9%), Fru + amantadine (+96.5%), Fru + aminoguanidine (+67.6%), Fru + ALA (+94.3%), Fru + NAC (+89%), as well as Fru + AA (+173.4%) compared to BSA. NFK fluorescence was meaningfully attenuated in Gal + aminoguanidine (−29.4%) and Gal + NAC (−16%) versus Gal. The content of NFK was significantly higher in Gal + AA (+137%) than in Gal alone. The parameter was effectively intensified in Gal (+88%), Gal + amantadine (+93.2%), Gal + aminoguanidine (+62.1%), Gal + ALA (+93.8%), Gal + NAC (+73.9%), as well as Gal + AA (+208.5%) compared to BSA. NFK content was markedly lower in GO + aminoguanidine (−50.4%), GO + ALA (−6%), GO + NAC (−55.2%), and also GO + AA (−21.1%) than in GO. The marker was substantially increased in GO (+578.4%), GO + amantadine (+608.5%), GO + aminoguanidine (+287%), GO + ALA (+613.1%), GO + NAC (+259.2%), as well as GO + AA (+456.6%) in comparison with BSA. The fluorescence of NFK was efficiently reduced in MGO + amantadine (−8.2%) and MGO + aminoguanidine (−40.2%) versus MGO. The biomarker was substantially elevated in MGO (+739.6%), MGO + amantadine (+679.2%), MGO + aminoguanidine (+442.7%), MGO + ALA (+708.3%), MGO + NAC (+733.9%), and MGO + AA (+826.2%) compared to BSA. The biomarker was considerably lower in ChT + aminoguanidine (−33.9%), ChT + NAC (−29.2%), and ChT + AA (−30%) than in ChT. NFK fluorescence was relevantly elevated in ChT (+42.7%), ChT + amantadine (+45.5%), as well as ChT + ALA (+43%) in comparison with BSA (Figure 4).

The content of DT was significantly reduced in Glc + aminoguanidine (−14.6%) and Glc + NAC (−8.3%) compared to Glc. The biomarker was effectively elevated in Glc + AA (+32%) versus Glc. The fluorescence of DT was markedly higher in Glc (+51.1%), Glc + amantadine (+51.9%), Glc + aminoguanidine (+43.6%), Glc + ALA (+52.3%), Glc + NAC (+46.9%), as well as Glc + AA (+67.4%) in comparison with BSA. The parameter was substantially decreased in Fru + aminoguanidine (−30%) when compared to Fru. DT fluorescence was efficiently increased in Fru + AA (+28.4%) versus Fru alone. The marker was relevantly enhanced in Fru (+59.9%), Fru + amantadine (+59.6%), Fru + aminoguanidine (+42%), Fru + ALA (+60.7%), Fru + NAC (+59.4%), as well as Fru + AA (+77%) compared to BSA. DT content was markedly attenuated in Gal + aminoguanidine (−23.5%) versus Gal. The fluorescence of DT was significantly higher in Gal + AA (+49%) than in Gal alone. The parameter was effectively increased in Gal (+80.4%), Gal + amantadine (+83.4%), Gal + aminoguanidine (+61.5%), Gal + ALA (+86.4%), Gal + NAC (+75.5%), as well as Gal + AA (+119.8%) compared to BSA. DT fluorescence was markedly lower in GO + aminoguanidine (−50.8%), GO + NAC (−54.9%), and also GO + AA (−23.6%) than in GO. DT content was significantly increased in GO + ALA (+8.6%) versus GO alone. The marker was substantially elevated in GO (+148.7%), GO + amantadine (+155.7%), GO + aminoguanidine (+73.1%), GO + ALA (+161.5%), as well as GO + AA (+113.7%) in comparison with BSA. DT content was effectively reduced in MGO + amantadine (−4.8%) and MGO + aminoguanidine (−54.3%) versus MGO. The biomarker was substantially raised in MGO (+291.8%), MGO + amantadine (+277.9%), MGO + aminoguanidine (+133.5%), MGO + ALA (+283.2%), MGO + NAC (+273.2%), as well as MGO + AA (+319.3%) compared to BSA. The biomarker was significantly lower in ChT + aminoguanidine (−37.2%), ChT + NAC (−13.6%), and also ChT + AA (−25.7%) than in ChT. DT content was relevantly elevated in ChT (+10.1%), ChT + amantadine (+11.4%), ChT + aminoguanidine (+6.4%), ChT + ALA (+10.4%), as well as ChT + AA (+7.5%) in comparison with BSA (Figure 4).

### Protein glycation products

The Maillard reaction is a series of chemical transformations that occur between amino acids and reducing sugars. Glycation is initiated by the covalent attachment of reducing sugars to the amino groups of proteins to produce a reversible and unstable Schiff base. The Schiff base can be converted into more stable AP which undergo dehydration and rearrangement accompanied by the formation of AGE, such as N^ϵ^-carboxymethyl-lysine (CML) and N^ϵ^-carboxyethyl-lysine (CEL). Prolonged exposure of proteins to Glc and other sugars causes the α-helix transition to a linear structure, triggering the formation of βA^82–84^.

#### Amadori products (AP)

**Figure 5.**
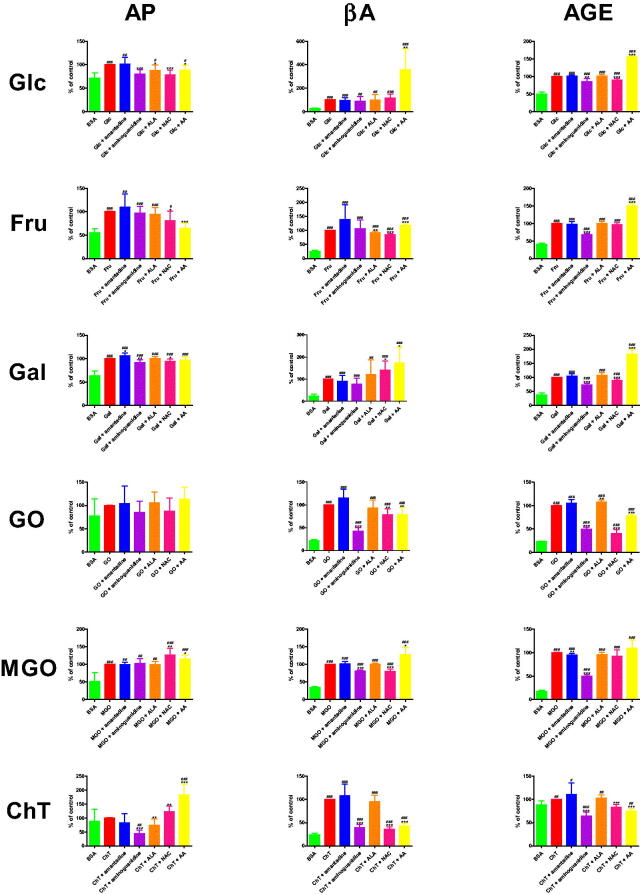
The influence of amantadine and other additives on protein glycation products in various models. AA: ascorbic acid; AGE: advanced glycation end products; ALA: α-lipoic acid; AP: Amadori products; BSA: bovine serum albumin; βA: β-amyloid; ChT: chloramine T-induced albumin oxidation; Fru: fructose-induced albumin glycation; Gal: galactose-induced albumin glycation; Glc: glucose-induced albumin glycation; GO: glyoxal-induced albumin glycation; MGO: methylglyoxal-induced albumin glycation; NAC: N-acetylcysteine; ∗*p* < 0.05 versus positive control (glycation/oxidising agent); ∗∗*p* < 0.01 versus positive control (glycation/oxidising agent); ∗∗∗*p* < 0.001 versus positive control (glycation/oxidising agent); ^#^*p* < 0.05 versus negative control (BSA); ^##^*p* < 0.01 versus negative control (BSA); ^###^*p* < 0.001 versus negative control (BSA).

The concentration of AP was significantly lower in Glc + aminoguanidine (−19.9%), Glc + ALA (−12.5%), Glc + NAC (−21.9%), and Glc + AA (−11.4%) than in Glc. The biomarker was effectively elevated in Glc (+39.5%), Glc + amantadine (+40%), Glc + ALA (+34.6%), as well as Glc + AA (+35%) in comparison with BSA. AP level was markedly decreased in Fru + NAC (−19.2%) and also Fru + AA (−35.1%) versus Fru. The parameter was effectively elevated in Fru (+82.4%), Fru + amantadine (+90.2%), Fru + aminoguanidine (+80.2%), Fru + ALA (+78.1%), and Fru + NAC (+66.6%) in comparison with BSA. The concentration of AP was markedly enhanced in Gal + amantadine (+6.3%) versus Gal. The marker was significantly reduced in Gal + aminoguanidine (−8.5%) and Gal + NAC (−5.6%) compared to Gal alone. The level of AP was relevantly higher in Gal (+57.5%), Gal + amantadine (+61.2%), Gal + aminoguanidine (+52.7%), Gal + ALA (+57.7%), Gal + NAC (+54.3%), and Gal + AA (+55.8%) than in BSA. This parameter was markedly raised in MGO + NAC (+26.7%) and MGO + AA (+15%) in comparison with MGO. The concentration of AP was considerably increased in MGO (+96%), MGO + amantadine (+95.6%), MGO + aminoguanidine (+98.2%), MGO + ALA (+95.4%), MGO + NAC (+121.6%), as well as MGO + AA (+110.4%) when compared to BSA. AP formation was markedly inhibited in ChT + aminoguanidine (−56.2%) and ChT + ALA (−25.4%) versus ChT. AP level was relevantly higher in ChT + NAC (+22.7%) and ChT + AA (+82.3%) than in ChT alone. The parameter was significantly enhanced in ChT + aminoguanidine (+5.2%), as well as ChT + AA (+21.8%) in comparison with BSA (Figure 5).

#### *β*−*amyloid (βA)*

βA fluorescence was effectively elevated in Glc + AA (+259%%) versus Glc. The biomarker was markedly higher in Glc (+302.1%), Glc + amantadine (+286%), Glc + aminoguanidine (+271.5%), Glc + ALA (+300%), Glc + NAC (+355%), as well as Glc + AA (+1086.9%) in comparison with BSA. The content of βA was substantially decreased in Fru + ALA (−7.6%) and Fru + NAC (−14.5%) compared to Fru. This parameter was markedly increased in Fru + AA (+17.7%) versus Fru alone. The marker was relevantly enhanced in Fru (+283.7%), Fru + amantadine (+394.4%), Fru + aminoguanidine (+301.3%), Fru + ALA (+262.1%), Fru + NAC (+242.5%), as well as Fru + AA (+334%) compared to BSA. The fluorescence of βA was significantly higher in Gal + NAC (+40.3%) and Gal + AA (+74%) than in Gal. The parameter was effectively elevated in Gal (+326.6%), Gal + amantadine (+296.9%), Gal + aminoguanidine (+254.5%), Gal + ALA (+391.9%), Gal + NAC (+457.9%), and Gal + AA (+568.3%) when compared to BSA. βA content was markedly lower in GO + aminoguanidine (−57.9%), GO + NAC (−22%), and GO + AA (−21.8%) than in GO. The production of βA was substantially increased in GO (+363.6%), GO + amantadine (+417.8%), GO + aminoguanidine (+153%), GO + ALA (+338.4%), GO + NAC (+283.7%), as well as GO + AA (+284.3%) in comparison with BSA. βA fluorescence was efficiently reduced in MGO + aminoguanidine (−19%) and MGO + NAC (−20.2%) versus MGO. This parameter was meaningfully higher in MGO + AA (+27.2%) than in MGO alone. The content of βA was substantially raised in MGO (+190.4%), MGO + amantadine (+193.4%), MGO + aminoguanidine (+154.2%), MGO + ALA (+191.3%), MGO + NAC (+151.9%), and MGO + AA (+242.1%) versus BSA. The marker was efficiently lower in ChT + aminoguanidine (−60.2%), ChT + NAC (−63.8%), and ChT + AA (−57%) than in ChT. βA fluorescence was relevantly elevated in ChT (+322.8%), ChT + amantadine (+348.3%), ChT + aminoguanidine (+128.5%), ChT + ALA (+306.4%), ChT + NAC (+116.9%), as well as ChT + AA (+138.8%) in comparison with BSA (Figure 5).

#### Advanced glycation end products (AGE)

The content of AGE was significantly reduced in Glc + aminoguanidine (−15.2%) and Glc + NAC (−10%) compared to Glc. AGE production was effectively elevated in Glc + AA (+55.5%) versus Glc alone. AGE fluorescence was markedly higher in Glc (+100.2%), Glc + amantadine (+101.4%), Glc + aminoguanidine (+85%), Glc + ALA (+101%), Glc + NAC (+90.1%), as well as Glc + AA (+155.7%) in comparison with BSA. The parameter was substantially decreased in Fru + aminoguanidine (−31.9%) than in Fru. AGE content was significantly increased in Fru + AA (+49.7%) versus Fru alone. The marker was relevantly enhanced in Fru (+144.4%), Fru + amantadine (+140.2%), Fru + aminoguanidine (+98.3%), Fru + ALA (+144%), Fru + NAC (+139.3%), and Fru + AA (+216.1%) compared to BSA. The fluorescence of AGE was considerably attenuated in Gal + aminoguanidine (−27.3%) and Gal + NAC (−10.8%) versus Gal. This parameter of AGE was significantly higher in Gal + AA (+82.6%) than in Gal alone. The content of AGE was effectively increased in Gal (+163.9%), Gal + amantadine (+170.6%), Gal + aminoguanidine (+119.2%), Gal + ALA (+176.2%), Gal + NAC (+146.3%), as well as Gal + AA (+299.3%) compared to BSA. AGE fluorescence was markedly lower in GO + aminoguanidine (−50.5%), GO + NAC (−59.9%), and GO + AA (−22.4%) than in GO. The biomarker was substantially enhanced in GO + ALA (+7.5%) versus GO alone. AGE content efficiently increased in GO (+333.5%), GO + amantadine (+351.1%), GO + aminoguanidine (+165%), GO + ALA (+358.5%), GO + NAC (+133.8%), and GO + AA (+258.7%) in comparison with BSA. The parameter was relevantly reduced in MGO + amantadine (−5.1%) and MGO + aminoguanidine (−50.5%) versus MGO. The generation of AGE was significantly raised in MGO (+471.4%), MGO + amantadine (+447.5%), MGO + aminoguanidine (+233.4%), MGO + ALA (+450.5%), MGO + NAC (+437.5%), as well as MGO + AA (+514.8%) compared to BSA. The fluorescence of AGE was significantly lower in ChT + aminoguanidine (−35.7%), ChT + NAC (−16.4%), and ChT + AA (−25.7%) than in ChT. This parameter was effectively elevated in ChT (+13.5%), ChT + amantadine (+15%), ChT + aminoguanidine (+8.7%), ChT + ALA (+13.9%), as well as ChT + AA (+10.1%) in comparison with BSA (Figure 5).

### Validation

Since fluorometric measurements of BSA glycoxidation can be interfered by additives, AGE content was also determined by ELISA. We showed that AGE evaluation by the fluorometric technique is consistent with the results of the reference method (ELISA) (Figure S1).

### Protein oxidation products

PC are generated by the oxidation of amino acids having free amino, amide or hydroxyl groups (e.g. arginine (ARG), lysine (LYS) and TRY). AOPP are the end products of the complex process of protein oxidation. They are aggregates, fragments or derivatives of oxidatively altered albumin, fibrinogen or lipoproteins. It is known that AOPP molecules contain DT, PC, and modified residues of TRY, TYR, ARG, LYS and sulphur-containing amino acids^85^^,^^86^.

#### Protein carbonyls (PC)

**Figure 6.**
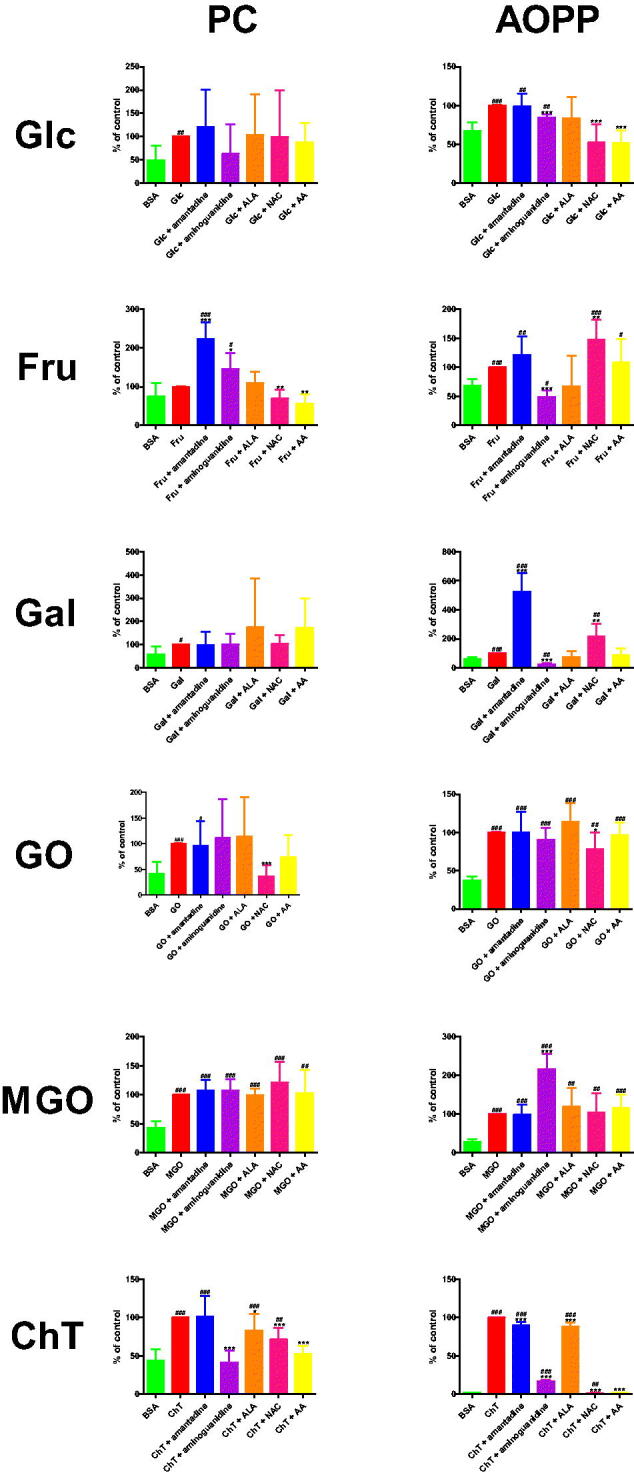
The influence of amantadine and other additives on protein oxidation products in various models. AA: ascorbic acid; ALA: α-lipoic acid; AOPP: advanced oxidation protein products; BSA: bovine serum albumin; ChT: chloramine T-induced albumin oxidation; Fru: fructose-induced albumin glycation; Gal: galactose-induced albumin glycation; Glc: glucose-induced albumin glycation; GO: glyoxal-induced albumin glycation; MGO: methylglyoxal-induced albumin glycation; NAC: N-acetylcysteine; PC: protein carbonyls; ∗*p* < 0.05 versus positive control (glycation/oxidising agent); ∗∗*p* < 0.01 versus positive control (glycation/oxidising agent); ∗∗∗*p* < 0.001 versus positive control (glycation/oxidising agent); ^#^*p* < 0.05 versus negative control (BSA); ^##^*p* < 0.01 versus negative control (BSA); ^###^*p* < 0.001 versus negative control (BSA).

PC concentration was significantly improved in Glc (+104.8%) in comparison to BSA. The biomarker was effectively increased in Fru + amantadine (+123.9%) and Fru + aminoguanidine (+45.8%) versus Fru. The level of PC was markedly decreased in Fru + NAC (−30.5%) and Fru + AA (−43.8%) compared to Fru alone. The parameter was substantially elevated in Fru + amantadine (+77.5%), as well as Fru + aminoguanidine (+50.5%) versus BSA. PC level was considerably higher in Gal (+71.4%) than in BSA. The marker was relevantly reduced in GO + NAC (−63.6%) in comparison with GO. The content of PC was markedly increased in GO (+142.3%) and GO + amantadine (+136.5%) compared to BSA. This parameter was significantly elevated in ChT (+136.9%), ChT + amantadine (+146.8%), ChT + aminoguanidine (+147.1%), ChT + ALA (+135.3%), ChT + NAC (+165.2%), as well as ChT + AA (+140.8%) versus ChT. PC concentration was substantially lower in ChT + aminoguanidine (−58.7%), ChT + ALA (−17.4%), ChT + NAC (−28.7%), and ChT + AA (−47.4%) than in ChT alone. The level of PC was markedly enhanced in ChT (+128.3%), ChT + aminoguanidine (+129.6%), ChT + ALA (+106%), as well as ChT + NAC (+91.5%) in comparison with BSA (Figure 6).

#### Advanced oxidation protein products (AOPP)

The level of AOPP was significantly lower in Glc + aminoguanidine (−15.5%), Glc + NAC (−47.8%), and Glc + AA (−48.3%) than in Glc. The biomarker was effectively improved in Glc (+48.5%), Glc + amantadine (+48%), as well as Glc + aminoguanidine (+41%) in comparison with BSA. AOPP concentration was markedly decreased in Fru + aminoguanidine (−51.5%) versus Fru. The parameter was significantly increased in Fru + NAC (+47.9%) compared to Fru alone. AOPP level was markedly elevated in Fru (+45.4%), Fru + amantadine (+55.2%), Fru + aminoguanidine (+22%), Fru + NAC (+67.1%), as well as Fru + AA (+49.1%) in comparison with BSA. The concentration of AOPP was considerably enhanced in Gal + amantadine (+424.8%) and Gal + NAC (+118.5%) versus Gal. The level of AOPP was significantly reduced in Gal + aminoguanidine (−75.8%) when compared to Gal alone. AOPP were relevantly higher in Gal (+67%), Gal + amantadine (+351.5%), Gal + aminoguanidine (+16.2%), and Gal + ALA (+146.3%) than in BSA. The parameter was markedly diminished in GO + NAC (−21.7%) in comparison with GO. The marker was significantly elevated in GO (+166.9%), GO + amantadine (+167.2%), and GO + aminoguanidine (+151.5%), GO + ALA (+190.7%), GO + NAC (+130.6%), as well as GO + AA (+161.1%) compared to BSA. AOPP concentration was notedly boosted in MGO + aminoguanidine (+115.8%) in comparison with MGO. This parameter was substantially increased in MGO (+249.8%), MGO + amantadine (+245.5%), MGO + aminoguanidine (+539%), MGO + ALA (+298.5%), MGO + NAC (+260.8%), as well as MGO + AA (+291%) when compared to BSA. AOPP level was markedly inhibited in ChT + amantadine (−10.2%), ChT + aminoguanidine (−82.6%), ChT + ALA (−11.9%), ChT + NAC (−98.4%), and ChT + AA (−98.1%) versus ChT. The concentration of AOPP was significantly enhanced in ChT (+4201.1%), ChT + amantadine (+3773.4%), ChT + aminoguanidine (+732.7%), ChT + ALA (+3701.1%), as well as ChT + NAC (+68.1%) in comparison with BSA (Figure 6).

### Binding affinity analysis

**Figure 7.**
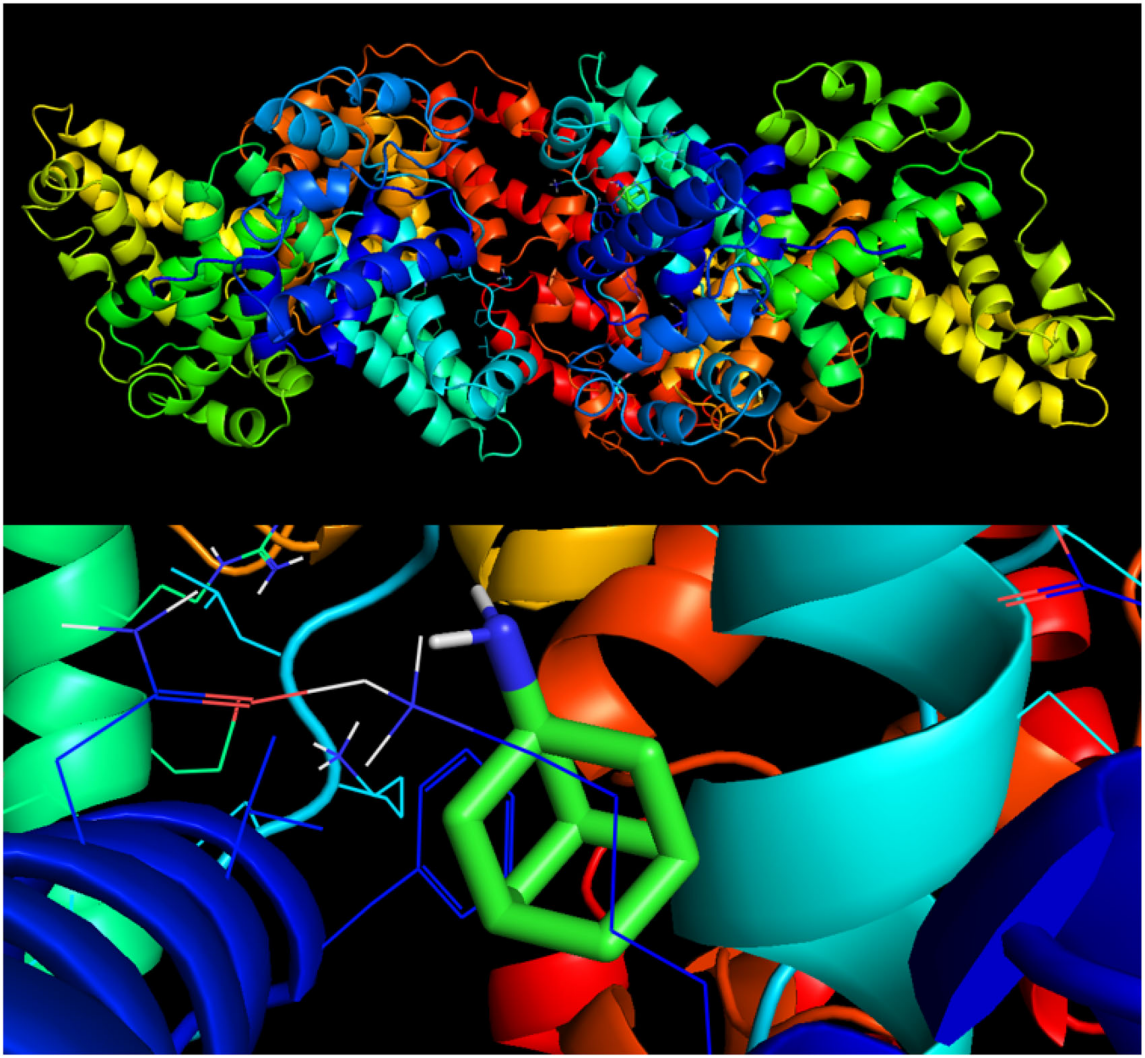
Visualisation of an amantadine docking site (mode 1) in BSA.

The simulation of molecular docking of amantadine exhibited its low affinity to a BSA particle with a score of −6.4 kcal/mol. Only four docking sites had root-mean-square deviations of atomic positions (RMSD) below 3 (Table 3), but none of them revealed any polar contact with whichever side chain of the BSA particle. Mode 1 is presented in Figure 7.

**Table 3.** The results of a molecular docking simulation of amantadine to BSA.

Mode	Affinity (kcal/mol)	RMSD (lower bond)	RMSD (upper bond)	Amino acid residues
1	−6.4	0.000	0.000	
2	−6.4	0.023	2.271	
3	−6.4	0.130	2.297	
4	−6.4	1.455	2.999	
5	−5.6	33.261	34.494	TYR-160
6	−5.6	33.245	34.455	TYR-160
7	−5.6	33.257	34.513	
8	−5.6	33.528	34.787	LEU-115
9	−5.6	33.514	34.765	LEU-115

*Note:* LEU: leucine; RMSD: root-mean-square deviations of atomic positions; TYR: tyrosine.

## Discussion

Amantadine is a drug used in the prevention/treatment of influenza A, but primarily in Parkinson’s disease and parkinsonian syndromes^87^. Despite its multidirectional effects, the exact mechanism of its pharmacological action is not well understood^88^^,^^89^. However, the potential antioxidant properties of amantadine have been reported in the literature^90–92^. Previous studies indicate that amantadine reduces the severity of systemic oxidative stress and stimulates non-enzymatic antioxidant defences (Table 2). This fact is not surprising since oxidative stress plays a vital role in the pathogenesis of both neurodegenerative and viral diseases^93–95^. Amantadine-treated patients showed decreased malondialdehyde concentration, increased β-carotene level, and longer survival after only one week of therapy^42^. Amantadine demonstrated a protective impact against lipopolysaccharide (LPS) and 1-methyl-4-phenyl-1,2,3,6-tetrahydropyridine (MPTP) (dopaminergic neurotoxins) toxicity in rat midbrain cultures by inhibiting the release of proinflammatory factors in microglia, enhancing the astroglial expression of a glial-derived neurotrophic factor (GNDF), and reducing the activation of NOX (Phox)^36^. However, it is not known whether the amantadine antioxidant activity is due to the properties of the substance itself or to the action on the nervous system and the secondary reduction of oxidative/nitrosative stress. The antiglycation effect of amantadine is also unknown. Therefore, we performed a comprehensive *in vitro* study determining the impact of amantadine on protein glycoxidation. We used the BSA model for this purpose.

Albumin is the major plasma protein^96^. It is a key transport and buffering molecule, and due to its property of binding transition metal ions, it also demonstrates strong antioxidant potential^97^. BSA contains 35 thiol groups, 34 of which participate in disulphide bridge formation^98^. The final products of protein oxidation are AOPP – derivatives of modified albumin formed by the accumulation of oxidised ARG, DT, and TRY residues^99^. The process of protein oxidation occurs simultaneously with protein glycation whose first products are Schiff bases and AP, and the final products are AGE^100^. Some of glycation products exhibit fluorescent properties, which enables their determination even at very low concentrations. Thus, measuring modified amino acids (TRY, KN, NFK, and DT) and AGE by fluorimetry provides a reliable assessment of carbonyl stress in biological samples. Several studies have shown that the fluorescence of TRY, KN, NFK, DT and AGE highly correlates with their concentrations evaluated by means of the ELISA method^60^^,^^101^.

In the present study, we used various glycation (Glc, Fru, Gal, GO, and MGO) and oxidation (ChT) factors to determine the impact of amantadine on glycoxidation, glycation, and oxidation protein products. Glycation is a non-enzymatic reaction that occurs between the carbonyl group of reducing sugars and proteins with high levels of free amino groups^82^. We showed that the content of glycoxidation products was significantly higher in BSA samples with the addition of all glycation agents compared to BSA without additives (except TRY). Although incubation conditions and reagent concentrations were chosen based on previous kinetic studies of albumin glycoxidation^47–49^^,^^51^^,^^53^^,^^56–58^, this also confirms the utility of sugars and aldehydes as glycation agents in an *in vitro* BSA model. Two LYS residues (LYS-524 and LYS-232) were found to be the main sites of Glc-induced glycation of BSA responsible for about 30% of total albumin glycation^102^. Endogenous α-oxoaldehydes such as GO and MGO are also responsible for protein glycation. GO and MGO are direct precursors of CML and CEL, the most important products from the group of AGE^14^^,^^103^. ARG and histidine (HIS) are the favoured interaction sites for MGO, while LYS is less likely to react with MGO^104^. Oxidants such as ChT are also responsible for post-translational modifications of proteins. ChT causes oxidation of amino acid residues, fragmentation of the protein backbone, and formation of cross-links between amino acids or additional PC in its structure^105^. It was demonstrated that substances such as limonene, sinensetin, naringenin and eriodictyol prevent glycoxidation by stabilising the conformation of BSA^106–109^. Since fluorometric measurements of BSA glycoxidation can be interfered by additives^47^^,^^48^^,^^51^, AGE content in our study was also determined by ELISA. We showed that AGE evaluation by the fluorometric technique is consistent with the results of the reference method. This has also been confirmed by the outcomes of other studies^47^^,^^48^^,^^51^^,^^54^^,^^55^. Glycoxidation biomarkers in BSA treated with amantadine were generally not different from the control group (glycation/oxidation factors), indicating that the drug did not affect oxidation and glycation processes. In some cases, amantadine even showed glycoxidant (↓TRY in all models, ↑KN and ↑NFK in Glc), proglycation (↑AP in Gal), and prooxidant (↑AOPP in Glc) properties. Noteworthy are the fluorescence changes of TRY, since this amino acid plays a special role in the BSA structure. The attachment of a ligand molecule to the binding site reduces the fluorescence of TRY and directly translates into the ligand-binding capacity of albumin. The decrease in TRY fluorescence influenced by the glycation and oxidising agents is therefore not surprising. Glycation agents affect TRY quenching due to the partial opening of hydrophobic pockets in glycation-modified albumin^110^. Additionally, prolonged exposure of BSA to Glc and other sugars cause the α-helix transition to a linear structure, giving rise to βA formation^111^. However, amantadine does not affect TRY metabolism. Thereby the fluorescence of KN and NFK is also not reduced upon exposure to the drug.

Molecular docking analysis did not reveal strong binding sites of amantadine on the BSA structure, which can be a probable reason for the lack of protective effect against protein oxidation/glycation. The source of amantadine’s ability to promote oxidative and carbonyl stress may be another feature of its structure. The amino group attached to the cyclic ring is an activating substituent that may be the reason of prooxidant properties of the compound^112^. Indeed, the amino group is one of the most biologically active groupings. It significantly boosts the toxicity of many organic substances, which increases proportionally to the number of amino groups^113^. Thus, the unrecognised effects of amantadine may be related to the promotion of redox imbalance which stimulates endogenous adaptive mechanisms. Oxidant-induced strengthening of the antioxidant barrier is a primary defence mechanism against oxidative and carbonyl stress^114–116^. This is confirmed by previous studies (Table 2), but further research, both basic and clinical, is needed.

Well-known glycation inhibitors and antioxidants were used to compare amantadine’s ability to protect against carbonyl stress. Aminoguanidine prevents glycation through competition, dicarbonyl scavenging, as well as antioxidant activity due to the occurrence of a guanidinium group^117^. ALA or its reduced form, dihydro-lipoic acid, neutralises ROS and regenerates vitamin C and GSH, which, in turn, can utilise vitamin E^118^. NAC is a precursor of GSH and may act as a direct ROS-scavenger^119^. AA owes its antioxidant properties to its ability to donate electrons. Strong reducing properties of AA contribute to its reactivity towards ROS^120^. In this research, we showed that amantadine has significantly lower antioxidant and antiglycation effect than all ROS scavengers (ALA, NAC, AA) and protein glycation inhibitors (aminoguanidine). Although amantadine does not increase the total antioxidant activity (DPPH and TAC), it should be noted that the compound scavenges HO• and H_2_O_2_ at the level of about 50–65%. Amantadine can therefore exhibit antioxidant activity, although it is weaker than the commonly used antioxidants. Moreover, our study confirms previous reports on the proglycation properties of AA^51^. It is postulated that the ascorbyl radical contributes to protein glycation^51^^,^^121^.

The effects of other adamantane derivatives on carbonyl stress have never been comprehensively studied. The literature provides only isolated papers examining the antioxidant potential of these drugs. Nitroxyl radical derivatives of amantadine diminished the content of HO• and O_2_•^−^ in *in vitro* models, as well as reduced oxidative damage in 2-deoxyribose- and dopamine-secreting neurons^38^. Benzo[*b*]furan derivatives of amantadine inhibited lipid peroxidation *in vitro*^122^, similarly to TYR-amantadine^123^. In contrast, amantadine-derived phenolic azo Schiff bases showed no antioxidant activity^124^. While the antioxidant properties of memantine are supported by some reports (*in vitro* inhibition of ROS production and lipid peroxidation; in vivo enhancement of the antioxidant barrier and reduction of protein and lipid oxidation)^35^^,^^125–130^, data on glycation prevention remain inconclusive^130–133^. There are also no data on thromantadine, and the antiglycoxidative impact of rimantadine was not confirmed^134–136^. Therefore, further research is needed to explore the potential antiglycoxidative effects of both amantadine and its derivatives.

In summary, amantadine exerted poor antioxidant properties and a lack of antiglycation activity per se. Although amantadine poorly scavenged HO• and H_2_O_2_, it has significantly lower antioxidant and antiglycation effect than all ROS scavengers and protein glycation inhibitors. Data from the literature review indicate that the protective effect of amantadine is more likely due to improved neuronal metabolism, which is secondarily responsible for reduced protein oxidation and glycation^36^^,^^40^^,^^76^^,^^131^^,^^137^^,^^138^. Although our research will never replace animal studies or clinical trials, this is the first study to evaluate the antiglycation properties of amantadine. Further studies should be conducted to search for its hitherto unknown mechanisms of action.

## Supplementary Material

Supplemental Material

## Data Availability

The data that support the findings of this study are available from the corresponding author upon reasonable request. Some data may not be made available because of privacy or ethical restrictions.
