# Supplementary material for: Antiglycoxidative properties of amantadine – a systematic review and comprehensive in vitro study
Source: J Enzyme Inhib Med Chem. 2022 Nov 2;38(1):138–55. doi: 10.1080/14756366.2022.2137161 (PMC9639497; doi:10.1080/14756366.2022.2137161)
Supplement: Supplemental Material [file IENZ_A_2137161_SM3546.pdf]

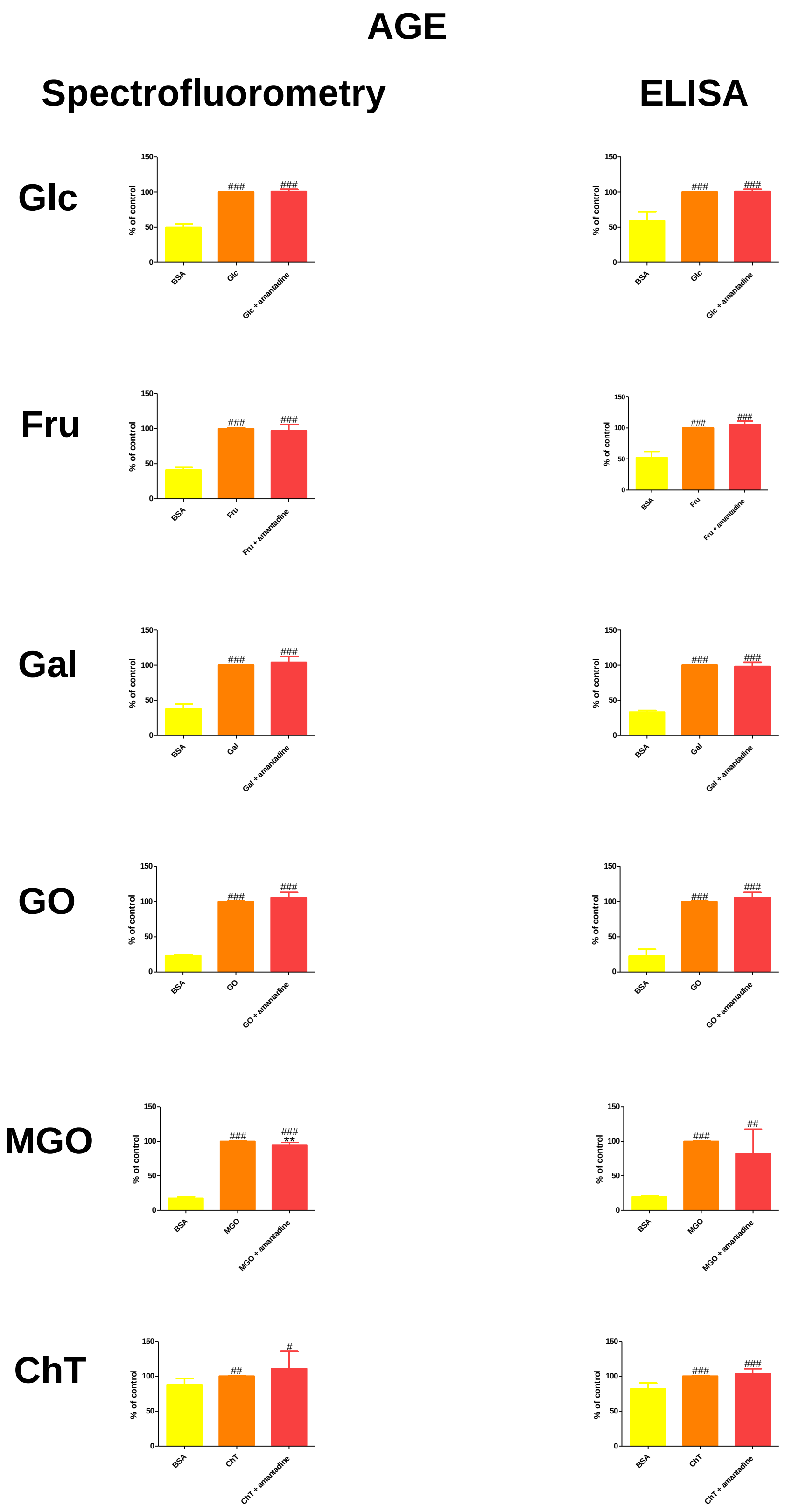

Figure S1. The influence of amantadine on AGE formation in various models measured spectrofluorometriclly and with the ELISA method.

AGE: advanced glycation end products; BSA: bovine serum albumin; ChT: chloramine T-induced albumin oxidation; ELISA: enzyme-linked immunosorbent assay; Fru: fructose-induced albumin glycation; Gal: galactose-induced albumin glycation; Glc: glucose-induced albumin glycation; GO: glyoxal-induced albumin glycation; MGO: methylglyoxal-induced albumin glycation; $**p < 0.01$ vs. positive control (glycation/oxidising agent); #$p < 0.05$ vs. negative control (BSA); ##$p < 0.01$ vs. negative control (BSA); ###$p < 0.001$ vs. negative control (BSA).
